# Negative regulation of lymphangiogenesis by Tenascin-C delays the resolution of inflammation

**DOI:** 10.1016/j.isci.2025.111756

**Published:** 2025-01-06

**Authors:** Daisuke Katoh, Yoshiyuki Senga, Kento Mizutani, Kazuaki Maruyama, Daishi Yamakawa, Tadashi Yamamuro, Michiaki Hiroe, Keiichi Yamanaka, Akihiro Sudo, Naoyuki Katayama, Toshimichi Yoshida, Kyoko Imanaka-Yoshida

**Affiliations:** 1 Department of Pathology and Matrix Biology, Mie University Graduate School of Medicine, Tsu, Mie, Japan; 2 Division of Endocrinology, Diabetes and Metabolism, Beth Israel Deaconess Medical Center and Harvard Medical School, Boston, MA, USA; 3 Department of Orthopedic Surgery, Mie University Graduate School of Medicine, Tsu, Mie, Japan; 4 Department of Dermatology, Mie University Graduate School of Medicine, Tsu, Mie, Japan; 5 Department of Physiology, Mie University Graduate School of Medicine, Tsu, Mie, Japan; 6 Department of Cardiology, National Center for Global Health and Medicine, Shinjuku-ku, Tokyo, Japan; 7 Department of Hematology and Oncology, Mie University Graduate School of Medicine, Tsu, Mie, Japan

**Keywords:** Natural sciences, Biological sciences, Immunology

## Abstract

Lymphatic vessels are required for the clearance of excess fluid and immune cells from inflamed tissue, making the regulation of lymphangiogenesis an important area of research. Although the positive regulatory mechanisms of lymphangiogenesis are well known, the negative regulatory mechanisms observed during inflammation remain unclear. Here, we identify tenascin-C (TNC) as a spatiotemporal negative regulator of lymphangiogenesis during inflammation. We found an inverse correlation between lymphangiogenesis and TNC expression in a mouse lymphedema model. Genetic deletion of *Tnc* promotes lymphangiogenesis and improves lymphatic drainage function, thereby accelerating the resolution of inflammation. Conversely, the exogenous addition of TNC suppresses lymphangiogenesis and prolongs inflammation. TNC inhibits the proliferation and promotes apoptosis of lymphatic endothelial cells. Mechanistically, TNC facilitates integrin αvβ1 heterodimer formation, leading to the activation of non-canonical (TAK1/p38MAPK/ATF-2) TGFβ signaling to suppress lymphangiogenesis. Our study highlights the importance of negative regulation of lymphangiogenesis in modulating immune responses.

## Highlights

• Tenascin-C spatiotemporally suppresses lymphangiogenesis, thereby sustaining inflammation
• Tenascin-C inhibits lymphangiogenesis through the integrin αvβ1/TGFβR/p38 MAPK pathway

## Graphical abstract

**Figure:**
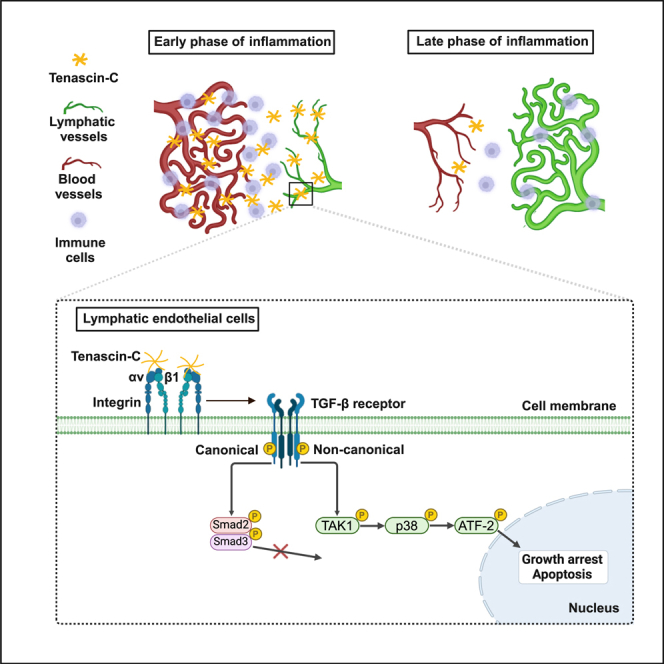

## Introduction

Inflammation is a complex biological process that is crucial for maintaining homeostasis in response to infection, tissue injury, or other external stimuli.^1^ The process of inflammation involves local and/or systemic responses, such as the migration and activation of leukocytes, and corresponding changes in the vasculature.^2^^,^^3^^,^^4^^,^^5^ Vascular angiogenesis, the growth of new blood capillaries, is crucial for delivering immune cells, oxygen, and nutrients to inflamed tissues, thereby initiating and sustaining immune responses.^4^ In contrast to vascular angiogenesis, lymphatic vessel angiogenesis —lymphangiogenesis— focuses on the removal of inflammatory cells and excess tissue fluid containing various cytokines and growth factors from inflamed tissues, which contributes to the resolution of inflammation.^2^^,^^3^^,^^5^ Both processes are important to the regulation of inflammation after injury. Importantly, lymphangiogenesis often occurs in the later stages of inflammation, while angiogenesis is predominant in its early stages.^6^^,^^7^^,^^8^^,^^9^ Intuitively, differences in the time courses of angiogenesis and lymphangiogenesis are expected given their respective roles; however, it raises an intriguing question as to why lymphangiogenesis follows angiogenesis during inflammation.

Extensive research has been conducted to identify the molecular drivers of lymphangiogenesis.^10^ Vascular endothelial growth factor C (VEGF-C) is a potent positive regulator of lymphangiogenesis,^11^^,^^12^^,^^13^ as it promotes the proliferation of lymphatic endothelial cells (LECs) through its receptor, vascular endothelial growth factor receptor 3 (VEGFR-3).^11^ The exogenous administration of VEGF-C ameliorates inflammatory disease by promoting lymphangiogenesis, while the inhibition of VEGFR-3 suppresses lymphangiogenesis therefore exacerbating inflammatory disease.^14^^,^^15^^,^^16^ In contrast, several factors, including TGF-β, have been reported to suppress the growth of lymphatic vessels^17^^,^^18^^,^^19^; however, the precise mechanisms responsible for the negative regulation of lymphangiogenesis in inflamed tissues remain unclear. A more detailed understanding of the regulatory mechanisms of lymphangiogenesis will offer novel insights for the development of innovative therapeutic strategies to manage inflammatory diseases.

The present study elucidates a mechanism by which lymphangiogenesis is negatively regulated during inflammation. Here, we identified tenascin-C (TNC), a matricellular protein,^20^^,^^21^ as a negative regulator of lymphangiogenesis. TNC was expressed at high levels during the early stages of inflammation in a strict spatiotemporal manner and regulated the growth of lymphatic vessels, which ultimately modulated the inflammatory response. TNC suppressed the growth of lymphatic vessels through non-canonical TGF-β signaling such as p38 MAPK.

## Results

### TNC is a candidate negative regulator of lymphangiogenesis during inflammation

**Figure 1:**
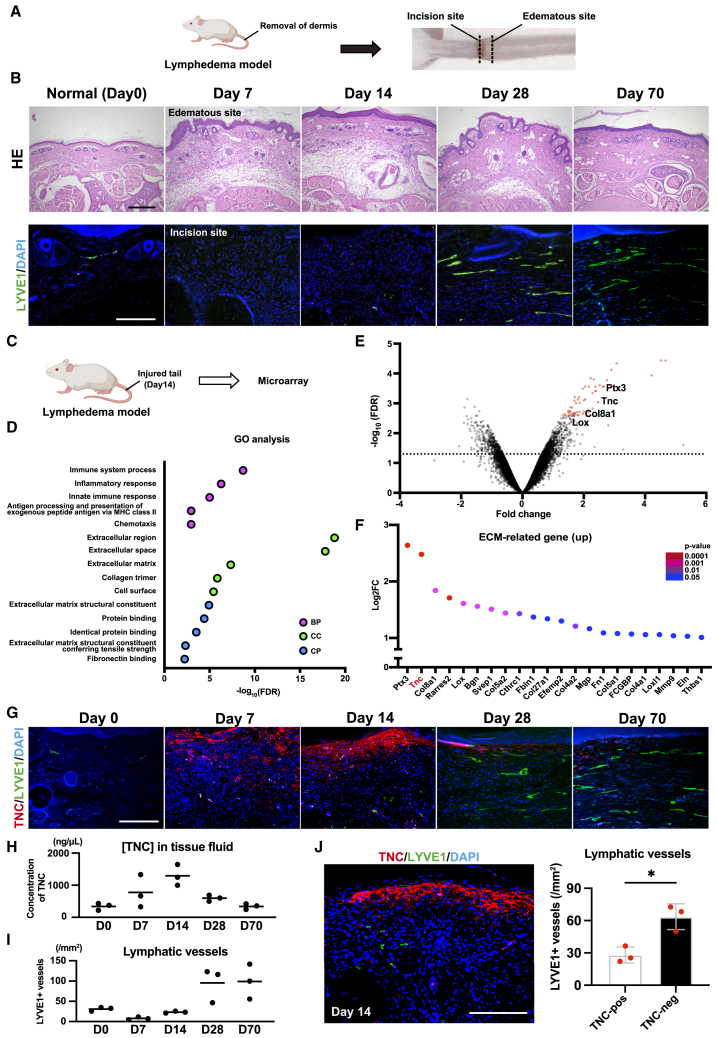
Identification of Tenascin-C as a candidate negative regulator of lymphangiogenesis (A and B) Analysis of lymphangiogenesis during inflammation using tail lymphedema mouse model. Schematic illustration of tail lymphedema mouse model (A), and histological analysis of tail lymphedema mouse model (B). Chronological changes in HE-stained tissue at edematous sites (upper panels) and immunostaining for LYVE1 (green; lymphatic vessel marker) at the incision site (lower panels) from day 0 (before surgery) to 70 days post-injury (dpi) (B). (C−F) Gene expression analysis of public microarray data (GSE4333) in tail lymphedema mouse model. Schematic illustration of sample collection for the microarray (C). Up-regulated biological pathways (BP), cellular components (CC), and cellular process pathways (CP) by a GO analysis of the microarray dataset (D) Volcano plot of microarray data (E), and relative expression levels of up-regulated extracellular matrix-related genes enriched in injured tail tissues relative to normal tail tissues (F). (G−J) Analysis of the relationship between TNC expression and lymphatic vessels in tail lymphedema mouse model. Chronological changes in immunofluorescent images of TNC (red) and LYVE1 (green) from day 0 (before surgery) to 70 dpi (G). A plot of the concentration of TNC in tail tissue fluid measured by ELISA (H), and the number of LYVE1+ lymphatic vessels in the tail tissues (I). *n* = 3 for each time point. A representative immunofluorescent image showing the spatial relationship between TNC deposition (red) and LYVE1+ lymphatic vessels (green) in the injured tail at 14 dpi (left) and a quantitative analysis of lymphatic vessels in TNC-positive and -negative areas (right) (J). *n* = 3. Each dot represents a value obtained from one sample. Data are presented as the means ± SD. Scale bars: 500 μm (B upper), 250 μm (B lower, G, and J). ∗*p* < 0.05 (unpaired t-test (I)).

To identify negative regulators of lymphangiogenesis during inflammation, we used tail lymphedema mouse model created by excising the tail epidermis, which contains abundant lymphatic vessels (Figure 1A). This model enables us to assess the relationships between lymphatic vessel growth and inflammation over time. We assessed the development of LYVE1-positive lymphatic vessels on the incision site, at which skin regeneration occurred, as well as inflammation and lymphedema at edematous sites, at which inflammatory cells accumulated (Figure 1B). We observed an abundant number of lymphatic vessels at 28- and 70-day post-injury (dpi), whereas we only detected a few lymphatic vessels at 7 and 14 dpi (Figure 1B). In contrast, the number of CD31-positive blood vessels was detected at 7 and 14 dpi, then decreased at 28 and 70 dpi (Figure S1). These results are consistent with previous findings that revealed angiogenesis preceded lymphangiogenesis during inflammation,^6^^,^^7^^,^^8^^,^^9^ indicating the presence of factors suppressing lymphangiogenesis around two weeks after injury in this model.

To identify candidate negative regulators of lymphangiogenesis, we reanalyzed microarray data (GSE4333) from normal tails and lymphedematous tails two weeks after injury (Figure 1C).^22^ Gene Ontology (GO) analyses of the microarray dataset revealed that differentially expressed genes (DEGs) in the tail lymphedema mouse model were enriched in the cellular component (CC) related to an extracellular region, the extracellular space, and extracellular matrix, and cellular process (CP) related to extracellular matrix components and extracellular matrix structural constituents conferring tensile strength (Figure 1D) (Table S1). This result is supported by previous findings showing that extracellular environmental components were important for tissue remodeling and enriched in inflammation.^23^ Consistent with the results of the GO analysis, several extracellular matrix (ECM)-related genes, including PTX3, TNC, and Col8a1, were significantly up-regulated in injured tail tissues (Figures 1E and 1F) (Table S2). TNC caught our attention, given its significant role in inflammation. TNC increases immediately after tissue injury to activate inflammatory responses and involves the persistence of inflammation.^24^^,^^25^^,^^26^^,^^27^

Therefore, we initially clarified the temporal expression and spatial localization of TNC molecules and lymphatic vessels in the tail lymphedema mouse model. To this end, we performed immunostaining for TNC in incision sites of the tail (Figure 1G) and measured TNC levels in tissue fluid from injured tails using an enzyme-linked immunosorbent assay (ELISA) (Figure 1H). TNC levels increased until 14 dpi and gradually decreased thereafter. The number of lymphatic vessels increased after the down-regulation of TNC expression (Figure 1I). Moreover, many of the newly developed lymphatic vessels were present in TNC-negative areas, while few were detected in TNC-positive areas (Figure 1J). In contrast, blood vessels appeared to grow in TNC-positive areas and increased with the up-regulation of TNC expression (Figures S1B and S1C). These results suggest that TNC functions as a spatial-temporal negative regulator of lymphangiogenesis during inflammation.

### Genetic deletion of TNC promotes the growth of lymphatic vessels and ameliorates lymphedema

**Figure 2:**
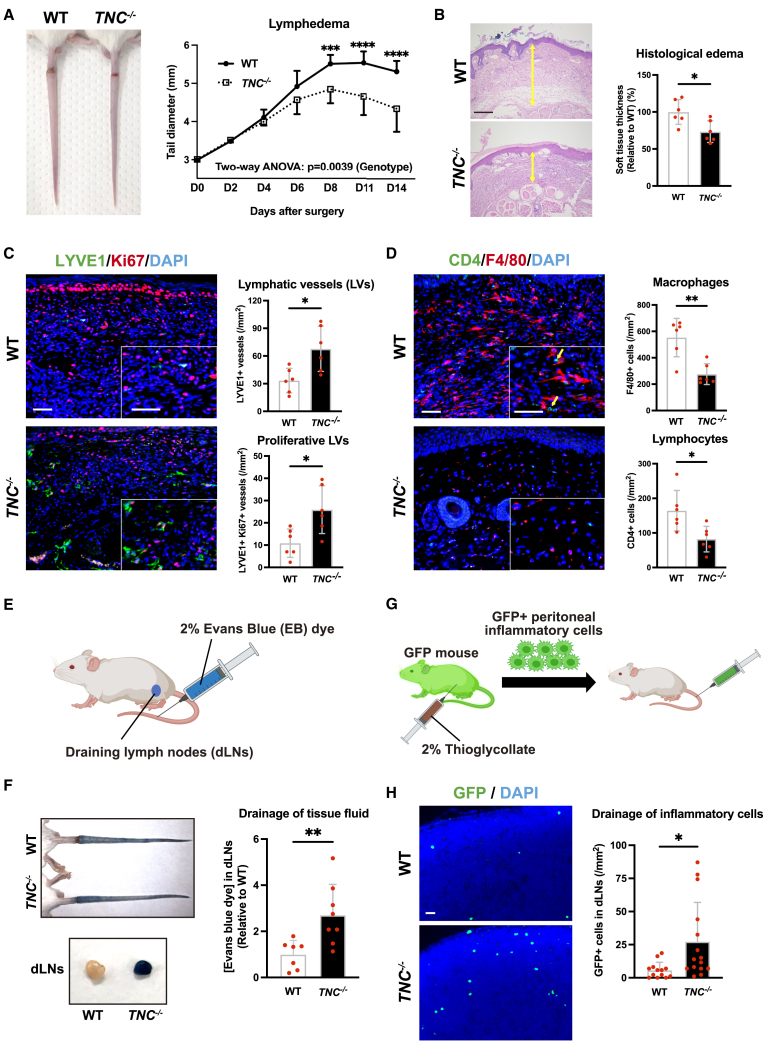
Genetic loss of tenascin-C promotes lymphangiogenesis and ameliorates lymphedema in mice (A and B) Comparison of the development of lymphedema in WT and *TNC*^*−/−*^ mice. A representative photo of the tail on day 14 (A, left) and a comparison of tail lymphedema and sequential changes in tail diameters between WT and *TNC*^*−/−*^ mice (A, right). *n* = 6 in each group. Comparison of the thickness of subcutaneous tissue at edematous sites (yellow arrows in photos) on day 14 (B). *n* = 6 in each group. (C and D) Comparison of the growth of lymphatic vessels and the infiltration of inflammatory cells in the injured tail tissues of WT and *TNC*^*−/−*^ mice. Representative immunofluorescence images of lymphatic vessels (LYVE1; green) and proliferative cells (Ki67; red) (C, left) and quantification of the number of lymphatic vessels and proliferative lymphatic vessels (C, right). Representative immunofluorescence images of macrophages (F4/80; red) and CD4^+^ lymphocytes (green) (yellow arrows) (D, left) and quantification of the number of macrophages and CD4^+^ lymphocytes (D, right). *n* = 6 in each group. (E and F) Assessment of the lymphatic drainage function of tissue fluid in WT and *TNC*^*−/−*^ mice. Schematic illustration of subcutaneous injection of Evans blue dye (EB) into the tail to assess the lymphatic drainage function of tissue fluid (E). Representative photos of blue-colored tails (F, left upper) and draining lymph nodes (dLNs) (F, left lower) 2 h after the injection. A comparison of EB concentrations in dLNs of WT and *TNC*^*−/−*^ mice (F, right). n = 7–8 in each group. (G and H) Assessment of the lymphatic drainage function of inflammatory cells in WT and *TNC*^*−/−*^ mice. Schematic illustration of subcutaneous injection of GFP+ peritoneal inflammatory cells, collected from thioglycollate-treated GFP+ mouse, into the tail (G) to assess the lymphatic drainage function of inflammatory cells. Representative immunofluorescent images of GFP (green) of dLNs 16 h after the injection (H, left), and quantification of GFP+ inflammatory cells in dLNs (H, right). *n* = 13–15 in each group. Each dot represents a value obtained from one sample. Data are presented as the means ± SD. Scale bars: 500 μm (B), 50 μm (C, D, and H). ∗*p* < 0.05, ∗∗*p* < 0.01, ∗∗∗*p* < 0.001, ∗∗∗∗*p* < 0.0001 (a two-way ANOVA (A) and unpaired t-test (B, C, D, F and H).

To determine whether TNC functions as a negative regulator of lymphangiogenesis and modulates inflammatory responses, we compared wild-type (WT) mice and TNC knock-out (*TNC* ^*−/−*^) mice in the tail lymphedema model. *TNC* ^*−/−*^ mice showed smaller tail diameters and a thinner dermis/epidermis than WT mice with tail lymphedema (Figures 2A and 2B). In other words, *TNC* ^*−/−*^ developed mild lymphedema. The number of proliferative newly developed lymphatic vessels, labeled by LYVE1 and Ki67, was significantly higher in *TNC* ^*−/−*^ mice than in WT mice (Figure 2C). Moreover, the number of inflammatory cells, including F4/80+ macrophages and CD4^+^ T lymphocytes, was significantly lower in *TNC* ^*−/−*^ mice (Figure 2D). Collectively, these results indicate that the genetic deletion of TNC promoted the growth of lymphatic vessels and ameliorated lymphedema as well as inflammation. In contrast to lymphatic vessels, the number of blood vessels was significantly lower in *TNC* ^*−/−*^ mice (Figure S1D).

Previous studies demonstrated that the promotion of lymphangiogenesis accelerated the resolution of inflammation^14^^,^^16^ because lymphatic vessels are crucial for the transport of tissue fluid or inflammatory cells from inflamed tissues to draining lymph nodes (dLNs) to attenuate inflammation.^2^^,^^3^^,^^5^ Therefore, we examined differences in the lymphatic drainage function of tissue fluid and inflammatory cells in WT and *TNC* ^*−/−*^ mice. To assess the lymphatic fluid drainage function of tissue fluid from inflamed tail tissues, we subcutaneously injected Evans Blue (EB) dye into the tail (Figure 2E). The dLNs of *TNC* ^*−/−*^ mice appeared to be dark blue, while those of WT mice were faintly blue (Figure 2F). Consistent with gross findings, the concentration of EB dye in dLNs was significantly higher in *TNC* ^*−/−*^ mice than in WT mice, indicating better lymphatic drainage function of tissue fluid in *TNC* ^*−/−*^ mice (Figure 2F). To assess the lymphatic drainage function of inflammatory cells, we collected GFP+ inflammatory cells from the peritoneal cavity of thioglycollate-treated GFP mice and injected them into the tails of WT and *TNC* ^*−/−*^ mice (Figure 2G). The number of GFP+ inflammatory cells in dLNs was significantly higher in *TNC* ^*−/−*^ mice than in WT mice (Figure 2H). These results revealed that the genetic deletion of TNC not only increased the number of lymphatic vessels but also enhanced the lymphatic drainage function of tissue fluid and inflammatory cells for the resolution of inflammation.

### TNC acts as a negative regulator of inflammation-associated lymphangiogenesis

**Figure 3:**
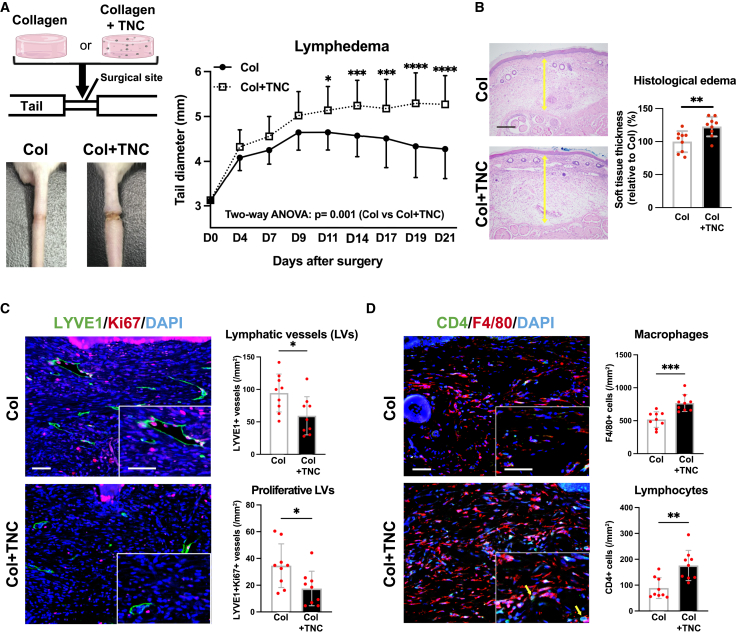
Exogenous administration of tenascin-C inhibits lymphangiogenesis and exacerbates lymphedema (A and B) Effects of the exogenous TNC treatment on the development of lymphedema in *TNC*^*−/−*^ mice. The incision sites of the tail were covered with collagen gels with or without TNC (A, left upper). A representative photo of the tail on day 21 (A, left lower) and sequential changes in tail diameters (A, right). *n* = 9 in each group. Comparison of the subcutaneous thickness at edematous sites (B, yellow arrows in photos) on day 21. *n* = 9 in each group. (C and D) Effects of the TNC treatment on the growth of lymphatic vessels and the infiltration of inflammatory cells with tail lymphedema in *TNC*^*−/−*^ mice. Representative immunofluorescence images of lymphatic vessels (LYVE1; green) and proliferative cells (Ki67; red) in tail tissues (C, left) and quantification of lymphatic vessels and proliferative lymphatic vessels (C, right). Representative immunofluorescence images of macrophages (F4/80; red) and CD4^+^ lymphocytes (yellow arrows) (CD4; green) (D, left), and quantification of the number of macrophages and CD4^+^ lymphocytes (D, right). *n* = 9 in each group. Each dot represents a value obtained from one sample. Data are presented as means ± SD. Scale bars: 500 μm (B), 50 μm (C and D). ∗*p* < 0.05, ∗∗*p* < 0.01, ∗∗∗*p* < 0.001, ∗∗∗∗*p* < 0.0001 (a two-way ANOVA (A) and unpaired t-test (B, C, and D).

The results obtained thus far indicate that TNC inhibited the growth of lymphatic vessels and prolonged inflammation. Therefore, we investigated whether an exogenous TNC treatment inhibits enhanced lymphangiogenesis in *TNC* ^*−/−*^ mice. Dermal incision sites were covered with a collagen gel containing TNC or not, followed by subcutaneous injection of TNC or PBS, respectively. As expected, the treatment with collagen gel containing TNC resulted in persistently larger tail diameters (Figure 3A) and a greater increase in the thickness of subcutaneous tissue (Figure 3B), indicating prolonged severe edema in TNC-treated mice. Furthermore, the collagen gel containing TNC decreased the number of newly developed lymphatic vessels (Figure 3C) and increased the number of infiltrating inflammatory cells (Figure 3D). These results revealed that TNC inhibited the growth of lymphatic vessels and prolonged inflammatory responses in the tail lymphedema model. Although inflammation is a common mechanism that protects the body from injury, the type of inflammatory response process varies depending on the initial stimulus or location in the body.^28^ We then investigated whether the negative regulation of lymphangiogenesis by TNC was limited in the tail lymphedema model or not. To this end, we created a zymosan-induced peritonitis model (Figure S2A). In this model, the number of lymphatic vessels in the diaphragm increased to remove pathogens and peritoneal fluid, while debris or inflammatory cells were detected at the peritoneal side of the diaphragm (Figure S2B). Consistent with the tail lymphedema model, the number of lymphatic vessels was significantly increased in *TNC* ^*−/−*^ mice (Figure S2C). However, no significant differences were observed in lymphatic vessels in various normal tissues between WT and *TNC* ^*−/−*^ mice (Figures S3A–S3F). Therefore, the function of TNC as a negative regulator of lymphangiogenesis may be limited in inflammation-associated pathological conditions.

### TNC inhibits the growth of LECs *in vivo* as well as *in vitro*

**Figure 4:**
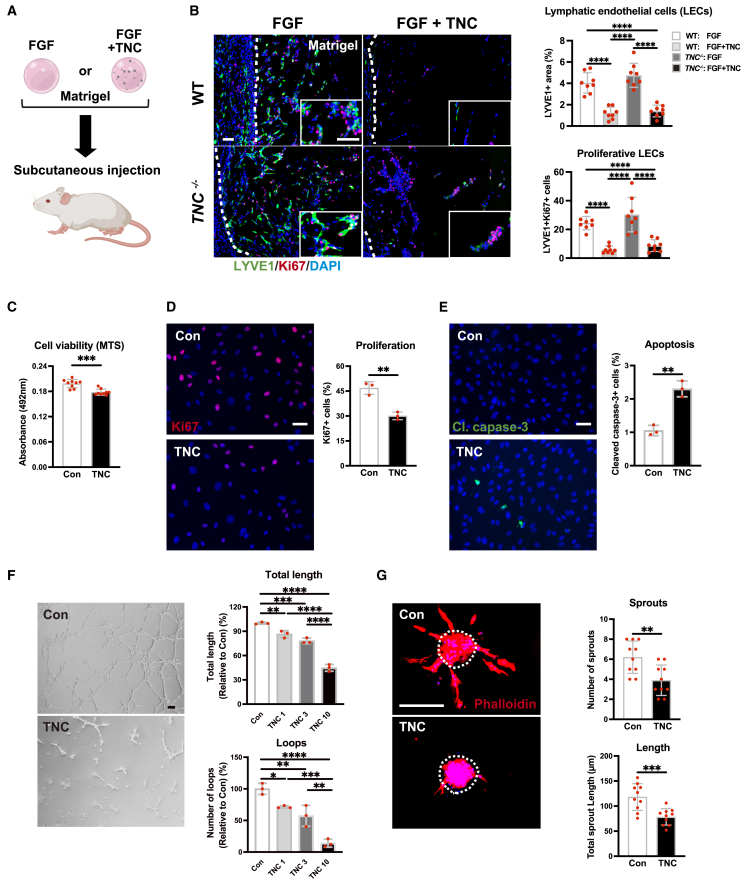
Tenascin-C inhibits the growth of lymphatic endothelial cells *in vivo* and *in vitro* (A and B) Analysis of the inhibitory effects of TNC on FGF-induced lymphangiogenesis. Schematic illustration of Matrigel plug assay using Matrigel containing FGF (4 μg/mL) alone or FGF with TNC (50 μg/mL) implanted into the subcutaneous of WT or *TNC*^*−/−*^ mice (A). Representative immunofluorescent images of lymphatic endothelial cells (LYVE1: green) and proliferative cells (Ki67: red) in Matrigel (B, left), and the quantification of LECs (B, right upper) and proliferative LECs (B, right lower). *n* = 8 in each group. (C–E) Effects of TNC on the viability, proliferation, and apoptosis of human dermal lymphatic endothelial cells (HDLECs) *in vitro*. Cell viability of HDLECs treated with or without TNC (10 μg/mL) for 48 h evaluated by the MTS assay (C). Representative immunofluorescent images of Ki67 (red) in HDLECs (D, left) and the quantification of proliferative cells (D, right). Representative immunofluorescent images of cleaved-caspase 3 (green) in HDLECs treated with or without TNC for 60 h (E, left) and the quantitative evaluation of apoptotic cells (E, right). (F and G) Effects of TNC on the tube formation and spheroid sprouting of HDLECs. Representative phase-contrasted images of HDLECs on Matrigel with or without TNC (10 μg/mL) for 16 h (F, left), a quantitative evaluation of the length of capillary-like structures (F, right upper), and the number of closed loops (F, right lower) under the treatment of various concentrations of TNC (1, 3, 10 μg/mL). Representative fluorescent images of rhodamine-phalloidin (red) in spheroids (left), measurement of the length of sprouts (G, upper), and the number of sprouts (G, lower). Each dot represents a value obtained from one sample. Data are presented as means ± SD. Scale bars: 500 μm (B), 50 μm (D–G). ∗*p* < 0.05, ∗∗*p* < 0.01, ∗∗∗*p* < 0.001 (a one-way ANOVA with the post-hoc Tukey’s test (B and F) or an unpaired t-test (C–E, G).

Several growth factors, including VEGF-C and FGF, are known to promote lymphangiogenesis *in vivo.*^29^^,^^30^ We herein investigated whether TNC inhibited lymphangiogenesis induced by these pro-lymphangiogenic factors, as observed in inflammation. To this end, we used a Matrigel assay and an ear sponge assay *in vivo*. Since we were hardly able to observe any lymphatic vessels in the absence of lymphangiogenic inducers (data not shown), we performed all the experiments under the treatment of FGF or VEGF-C. We subcutaneously implanted Matrigel containing FGF with or without TNC into WT and *TNC* ^*−/−*^ mice (Figure 4A). The numbers of LYVE1-positive lymphatic endothelial cells (LECs) and Ki67-positive proliferative LECs were significantly lower in Matrigel containing TNC in both WT and *TNC* ^*−/−*^ mice (Figures 4B and 4C). Next, we implanted a gelatin sponge containing VEGF-C with or without TNC into the ears of WT or *TNC* ^*−/−*^ mice (Figure S4A). Similar to the results of the FGF-Matrigel assay, we detected a lower number of LYVE1-positive lymphatic vessels in the sponge with VEGF-C and TNC than in the sponge with VEGF-C alone in both WT and *TNC* ^*−/−*^ mice (Figure S4B). Moreover, the number of proliferative lymphatic vessels was significantly lower in the sponge containing TNC (Figure S4C). These results demonstrated that TNC was able to inhibit lymphangiogenesis induced by the pro-lymphangiogenic factors, such as FGF and VEGF-C.

TNC regulates the survival of several cell types; however, responses to TNC vary depending on the cell type.^31^ A previous study reported that TNC increased the proliferation and tube formation ability of human dermal microvascular endothelial cells (HDMECs) *in vitro.*^32^ Since HDMECs contain both vascular and lymphatic endothelial cells, it remains unclear whether TNC increases or decreases the viability of LECs *in vitro.* To examine the effects of TNC on LECs *in vitro*, we used primary human dermal LECs (HDLECs) that do not contain vascular endothelial cells. Consistent with *in vivo* data, we found that TNC significantly decreased the viability of HDLECs *in vitro* (Figure 4C). We then investigated whether TNC inhibited proliferation and/or promoted apoptosis. Staining for the proliferative marker Ki67 and the apoptotic marker cleaved caspase-3 showed that TNC inhibited proliferation (Figure 4D) and promoted apoptosis in HDLECs (Figure 4E). Furthermore, TNC disturbed tube formation (Figure 4F) and the spheroid-based sprouting (Figure 4G) of HDLECs. These results revealed the direct negative effect of TNC on HDLECs.

### TNC increases the heterodimer formation of integrin αvβ1 and activates canonical and non-canonical TGF-β signaling

**Figure 5:**
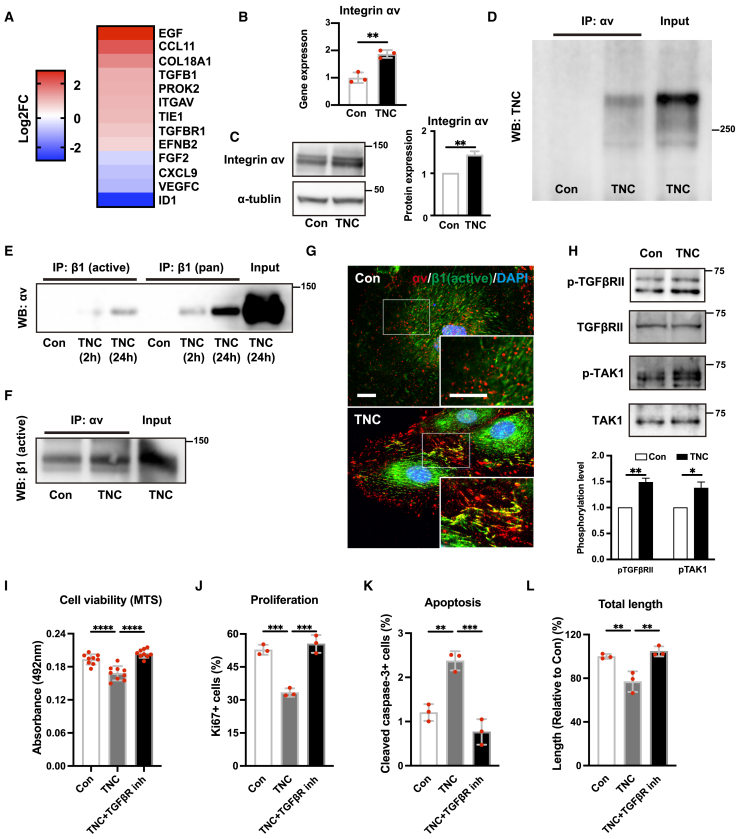
Tenascin-C increases heterodimer formation of integrin αvβ1 and activates TGF-β signaling (A) Heatmap of the micro-array transcriptome in HDLECs treated with or without TNC for 24 h. (B and C) Analysis of the expression of integrin αv in HDLECs treated with TNC. mRNA levels of integrin αv in HDLEC treated with or without TNC for 6 h determined by real-time PCR (B). Immunoblot analysis of integrin αv from HDLEC treated with or without TNC for 72 h (C). (D) Analysis of the interaction between integrin αv and TNC. Immunoblot analysis of TNC immunoprecipitated (anti-integrin αv) from HDLEC treated with or without TNC for 48 h. (E–G) Analysis of the interaction between integrin αv and β1. Immunoblot analysis of integrin αv immunoprecipitated (anti-integrin active β1 or pan β1) from HDLEC treated with or without TNC for 2 or 24 h (E). Immunoblot analysis of integrin β1 immunoprecipitated (anti-integrin αv) from HDLEC treated with or without TNC for 24 h (F). Representative immunofluorescence images of integrin αv (red) and integrin β1 (green) in HDLEC treated with TNC (G). (H) Immunoblotting of the phosphorylation of TGF-β receptor type II and TAK1 after the treatment with or without TNC for 1 h (upper) and their quantitative evaluation (lower). (I–L) Analysis of the effects of an inhibitor of the TGF-β receptor (LY2109761) on HDLECs treated with TNC. Quantification of cell viability evaluated by the MTS assay (I), the number of ki67-positive proliferative cells (J), the number of cleaved caspase-3 positive apoptotic cells (K), and tube formation ability by total tube length measurements (L) in HDLECs treated with TNC in the presence or absence of a TGF-β receptor inhibitor. Each dot represents a value obtained from one sample. Data are presented as means ± SD. Scale bars: 10 μm (G). ∗*p* < 0.05, ∗∗*p* < 0.01, ∗∗∗*p* < 0.001, ∗∗∗∗*p* < 0.0001 (an unpaired t-test (B and H) or a one-way ANOVA with the post-hoc Tukey’s test (I–L)).

Although we revealed the negative effects of TNC on lymphangiogenesis *in vivo* and *in vitro*, it is still unknown how TNC affects LECs mechanistically. To investigate the mechanisms by which TNC negatively affects LECs, we performed an angiogenesis-related microarray analysis of HDLECs treated with TNC. A transcriptome analysis showed that TNC up-regulated several genes in HDLECs, while the expression of FGF2 and VEGF-C was down-regulated (Figure 5A) (Table S3). The increased expression of integrin αv and TGF-β-related genes was of interest because we previously found that TNC bound integrin αvβ1 and transduced TGF-β signals.^33^^,^^34^ In addition, previous studies demonstrated the involvement of TGF-β signaling in suppressing the growth of LECs.^17^^,^^19^^,^^35^ We validated the up-regulated expression of integrin αv in HDLECs treated with TNC by PCR and immunoblotting (Figures 5B and 5C). Furthermore, immunoprecipitation assays showed that TNC was coprecipitated with integrin αv (Figure 5D), indicating the interaction of TNC and integrin αv. The integrin αv subunit forms a heterodimer with different several β subunits. Among them, integrin αvβ1, αvβ3, and αvβ6 are considered to be receptors for TNC.^36^ We focused on integrin αvβ1 because we previously showed that TNC bound to integrin αvβ1 in fibroblasts and activated TGF-β/SMAD signaling.^34^ Immunoprecipitation assays using the anti-pan-β1 and active β1 antibody revealed that the TNC treatment significantly increased the coprecipitation of αv subunit (Figure 5E). Immunoprecipitation assays using the anti- αv antibody showed that TNC also increased coprecipitation of the β1 subunit (Figure 5F). Double immunofluorescence demonstrated that the colocalization of integrin αv and β1 increased at focal adhesion sites in HDLECs treated with TNC (Figure 5G). Furthermore, TNC increased the phosphorylation of FAK and Src in HDLECs (data not shown). These results showed that TNC bound to and promoted the formation of the integrin αvβ1 heterodimer, which resulted in activation of the integrin signal cascade.

We also investigated whether TNC activated TGF-β signaling in HDLECs. Consistent with our previous findings on fibroblasts,^34^ TNC increased the phosphorylation of SMAD2/3 and TGFβ receptors (TGFβR) in HDLECs (Figures 5H and S5A). We also found that TNC increased the phosphorylation of TAK1, a key mediator of non-canonical TGFβ signaling (Figure 5H). To confirm whether TNC negatively affects HDLECs through TGF-β signaling, we examined the effects of a TGFβR inhibitor (LY2109761) or SMAD3 inhibitor (SIS3) on TNC-treated HDLECs. Inhibition of TGFβR decreased TNC-induced negative effects of HDLECs *in vitro* (Figures 5I–5L). However, inhibition of SMAD3 did not exert any of these effects (Figures S5B–S5E).

### TNC induces the phosphorylation of p38 MAPK via non-canonical TGF-β signaling

**Figure 6:**
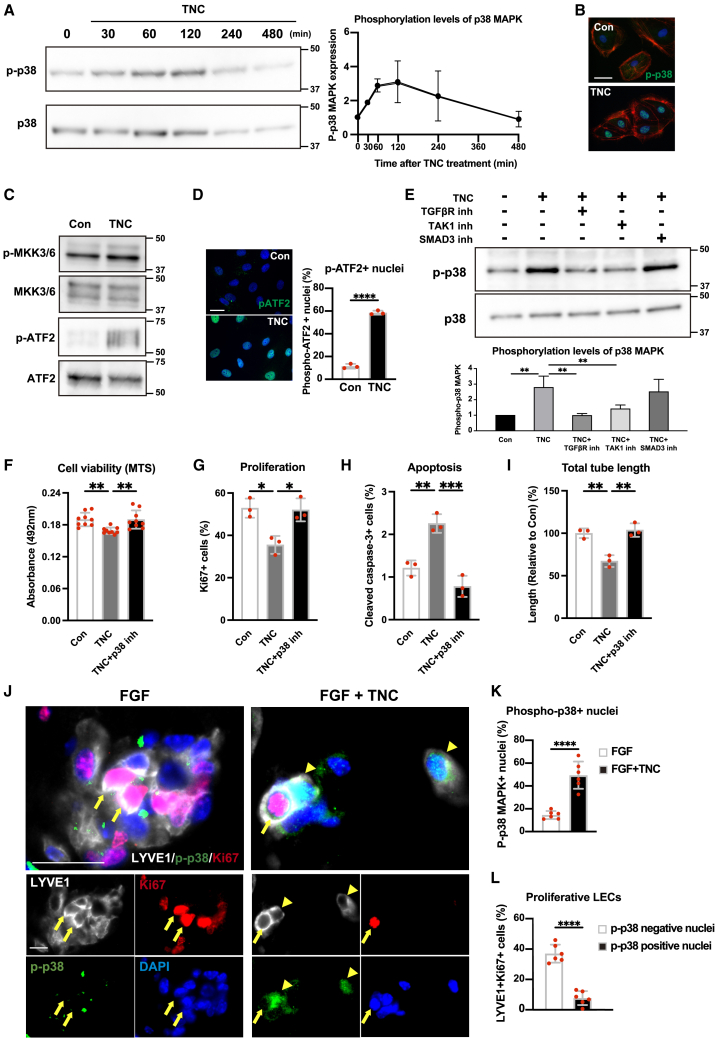
Tenascin-C negatively affects lymphatic endothelial cells through the p38 MAPK pathway (A and B) The effects of TNC on the phosphorylation of p38 MAPK in HDLECs. Representative images of the time course of immunoblotting for the phosphorylation of p38 MAPK and a quantitative analysis of HDLECs treated with TNC (A). Immunofluorescent staining of phosphorylated p38 MAPK (green), rhodamine-phalloidin, and DAPI in HDLECs (B). (C and D) The effects of TNC on p38 MAPK-related signaling molecules. An immunoblot analysis of the phosphorylation levels of MKK3/MKK6 or ATF2 in HDLECs (C). Representative fluorescent images of phospho-ATF2 (green) in HDLECs (D, left) and the quantification of phospho-ATF2-positive nuclei in HDLECs (D, right). (E) The effects of inhibitors of the TGF-β receptor (LY2109942; 2 μM), TAK1 (7-oz ((5Z)-7-Oxozeaenol); 0.1 μM), or SMAD3 (SIS3; 2 μM) on the TNC-induced phosphorylation of p38 MAPK. Representative Immunoblotting of phosphorylated p38 MAPK (E, upper) and its quantification (E, lower). (F–I) The effects of the p38 MAPK inhibitor (SB203580; 2μM) on TNC-induced negative effects in HDLECs. The quantification of cell viability evaluated by the MTS assay (F), the number of ki67 positive proliferative cells (G), the number of cleaved caspase-3 positive apoptotic cells (H), and tube formation ability by total tube length measurements (I) in HDLECs treated with TNC in the presence or absence of a p38 MAPK inhibitor. (J and K) Involvement of p38 MAPK phosphorylation in the regulation of lymphangiogenesis *in vivo*. Triple immunostaining for phospho-p38MAPK (green), ki-67 (red), and LYVE1 (white) in FGF-Matrigel with or without TNC implanted in mice (J). Representative images of nuclear p-p38-negative/Ki67-positive proliferating lymphatic endothelial cells (yellow arrows) and nuclear p-p38-positive/Ki67-negative lymphatic endothelial cells (yellow arrowheads). Quantification of the number of nuclear p-p38-positive lymphatic endothelial cells (K) and the number of Ki67-positive proliferative cells in p-p38-negative or p-p38-positive lymphatic endothelial cells (L). Each dot represents a value obtained from one sample. Data are presented as means ± SD, Scale bars: 10 μm (B and D), 50 μm (J). ∗*p* < 0.05, ∗∗*p* < 0.01, ∗∗∗*p* < 0.001, ∗∗∗∗*p* < 0.0001 (an unpaired t-test (D, K, and L), and a one-way ANOVA with the post-hoc Tukey’s test (E–I)).

The above results indicated that TNC affects HDLECs through SMAD-independent TGF-β signaling pathways. We focused on p38 mitogen-activated protein kinase (MAPK) signaling among the non-canonical TGF-β signaling pathways because p38 MAPK is a key regulator of cell survival as well as a downstream target of TAK1.^37^^,^^38^ As expected, TNC increased the phosphorylation of p38 MAPK, which peaked one to 2 h after the treatment (Figure 6A), and promoted the nuclear translocation of phospho-p38 MAPK in HDLECs (Figure 6B). TNC also induced the phosphorylation of mitogen-activated protein kinase kinase (MKK) 3/6, an upstream kinase of p38 MAPK, and the downstream transcriptional factor, activating transcription factor (ATF)-2, which regulates cell growth inhibition or apoptosis, and promoted the nuclear translocation of phospho-ATF2 (Figures 6C and 6D).^39^^,^^40^ We then investigated whether TGFβR/TAK1 signaling contributed to the phosphorylation of p38 MAPK in TNC-treated LECs. The inhibition of TGFβR or TAK1 suppressed the phosphorylation of p38 MAPK, while that of SMAD3 did not (Figure 6E). These results revealed that TNC phosphorylated p38 MAPK through the TGFβR/TAK1 pathway, but not the TGFβR/SMAD pathway. We also investigated the involvement of p38 MAPK in the inhibitory effects of TNC on HDLECs. An inhibitor of p38 MAPK (SB203580) ameliorated the TNC-induced negative effects of HDLECs (Figures 6F–6I). These results revealed that TNC activated p38 MAPK via non-canonical TGF-β signaling to inhibit cell growth and promote the apoptosis of HDLECs *in vitro*.

We clarified the importance of p38 MAPK signaling on TNC-induced negative effects on LECs *in vitro*. However, it is still unknown whether the p38 MAPK signaling involves the TNC-induced negative effects *in vivo*. To explore the relationships between TNC and phosphorylation of p38 MAPK *in vivo*, we examined lymphangiogenesis in the FGF-Matrigel assay by triple immunostaining for LYVE1, phospho-p38 MAPK (p-p38), and the proliferation marker Ki-67. The number of nuclear p-p38-positive LECs was significantly increased in Matrigel containing TNC (Figures 6J and 6K). In addition, more proliferative cells were observed in nuclear p-p38-negative LECs than in nuclear p-p38-positive (Figure 6L). These observations suggested that TNC induced the phosphorylation of p38 MAPK in LECs *in vivo*, and phosphorylation of p38 MAPK is also involved in regulating the growth of lymphatic vessels.

## Discussion

Key to the resolution of inflammation, lymphangiogenesis attracts considerable attention.^41^ However, the complete picture of the controlling mechanisms of lymphangiogenesis remains unclear. A careful observation of the lymphedema model revealed that lymphangiogenesis occurs late during inflammation, while angiogenesis occurs in the early phase. Therefore, we hypothesized that certain factors interfere with lymphatic vessel growth in the early phase of inflammation. We identified TNC as a negative regulator of lymphangiogenesis, which inhibits the growth and drainage function of lymphatic vessels and prolongs inflammation.

It is a mystery why lymphangiogenesis begins late in the inflammatory process, lagging behind angiogenesis, even though several pro-lymphangiogenic factors increase early in inflammation along with pro-angiogenic factors.^42^ TNC is a unique extracellular matrix molecule that is transiently expressed at specific sites in the early phase of inflammation and has diverse roles in tissue repair.^31^^,^^43^^,^^44^^,^^45^ TNC is known to act on a variety of cells, including macrophages or fibroblasts, to promote inflammation.^24^^,^^25^^,^^30^^,^^46^^,^^47^^,^^48^^,^^49^^,^^50^^,^^51^^,^^52^ Importantly, lymphangiogenesis increased after the reduction of TNC expression, while blood vessels increased along with increased TNC expression. In addition, lymphatic vessels are observed in the TNC-negative area, whereas blood vessels are observed in the TNC-positive area. Similar findings are observed in human cardiac tissues after infarction,^9^ indicating that TNC could be a negative regulator of lymphangiogenesis. Indeed, the genetic deletion of TNC promotes lymphangiogenesis and the exogenous administration of TNC suppresses lymphangiogenesis in inflammatory tissues. More interestingly, TNC inhibits the growth of lymphatic vessels induced by pro-lymphangiogenic factors *in vivo*. These observations partially explained why lymphatic vessels are not observed in the early phase of inflammation. TNC inhibits lymphangiogenesis and prevents the resolution of inflammation. In other words, TNC disturbs lymphangiogenesis to prolong the inflammatory response. This idea is supported by previous reports that showed that TNC contributes to prolonged inflammation and fibrosis.^47^ Thus, the present results suggest a mechanism by which TNC exacerbates and prolongs inflammation by suppressing lymphangiogenesis during the acute phase.

TNC transduces intracellular signals through a variety of cell surface receptors, such as integrins, depending on the cell type^31^^,^^36^. But until now it was unclear how TNCs negatively affect LECs. Several factors have been reported to negatively affect lymphangiogenesis, either directly or indirectly.^17^^,^^18^^,^^19^^,^^53^^,^^54^^,^^55^ TGF-β is one of the most studied anti-lymphangiogenic factors, that inhibits lymphangiogenesis in inflammatory and cancer tissues.^17^^,^^18^^,^^19^^,^^35^ Accumulated evidence suggests that TNC enhances TGF-β signaling.^34^^,^^56^^,^^57^^,^^58^^,^^59^ For example, TNCs bind integrin αvβ1 and activate TGF-β/SMAD signaling in fibroblasts to promote the conversion to myofibroblasts.^34^ Therefore, we speculate that TGF-β signaling may be involved in the anti-lymphangiogenic effects of TNC. Indeed, we found that TNC promoted the heterodimer formation of integrin αvβ1 in HDLECs, leading to the activation of the canonical pathway, TGF-βR/SMAD2/3, as well as the non-canonical pathway, TAK-1/p38 MAPK/ATF. Our inhibitory experiment revealed that the TAK1/p38 pathway, but not the SMAD pathway, is important. Collectively, these results suggest that the binding of TNC to integrin αvβ1 in LECs suppressed lymphangiogenesis by activating the TGFβ/TGF-βRII/TAK-1/p38 MAPK/ATF axis, as shown in the graphical abstract. On the other hand, our study didn’t explore how TNC affects classical lymphangiogenic signals including the VEGF-C/VEGFR3 pathway. Since we found that TNC inhibited FGF- or VEGF-C-induced lymphangiogenesis, it would be interesting to investigate whether TNC’s inhibitory effects are related to or independent of VEGF-C/VEGFR3 signaling.

In conclusion, we herein elucidated a novel negative regulatory mechanism of lymphangiogenesis during inflammation. A growing number of studies have shown that the administration of pro-lymphangiogenic factors, such as VEGF-C, reduces inflammation and inhibits disease progression. However, inflammation is an essential response during tissue repair after injury. A more detailed understanding of negative regulatory mechanisms may contribute to the development of innovative therapeutic strategies to control inflammation at an appropriate level by balancing pro- and anti-lymphangiogenic factors.

### Limitations of the study

One of the limitations of this study is it is difficult to create a mouse model in which the effects of TNC are specifically deleted in lymphatic endothelial cells. Therefore, our results from *in vivo* experiments reflect both the direct effects of TNC on lymphatic vessels and its indirect effects through other types of cells including inflammatory cells or blood vessels, although we assessed the direct impact of TNC on lymphatic vessels *in vitro*. Resolving this critical issue is necessary for the further advancement of ECM research.

## Resource availability

### Lead contact

Further information or requests for experimental data should be addressed to the lead contact, Daisuke Katoh (307028@gmail.com).

### Materials availability

This work did not generate new reagents.

### Data and code availability

• Data: All raw data in this paper are available from the lead contact, upon reasonable request.
• Code: This paper does not report original code.
• All other requests: Any additional information required to reanalyze the data reported in this paper is available from the lead contact upon reasonable request.

## Acknowledgments

The authors thank Miyuki Namikata and Mari Hara for their technical assistance. This work was supported in part by Grants-in-Aid for Scientific Research from the 10.13039/501100001691Japan Society for the Promotion of Science and from the Ministry of Education, Science, Technology, Sports and Culture of Japan (17K15615 and 21K15417 to D.K., 20K18029 and 22K16741 to Y.S., 19K07662 to T.Y., 18K06890 to K.I.-Y.).

## Author contributions

D.K. designed the research; D.K., Y.S., and K.M. performed the experiments and analyzed the data; K.M., D.Y., T.Y., M.H., K.Y., A.S., N.K., T.Y., and K.I.-Y. were involved in data interpretation and reviewed the manuscript; D.K., Y.S., T.Y., and K.I.-Y. acquired research funding. K.I.-Y. contributed valuable discussions. D.K. wrote the manuscript. D.K. is the guarantor of this work, has full access to all the data in the study, and takes responsibility for its integrity and the accuracy of the data analysis.

## Declaration of interests

T.Y. receives royalties on TNC antibodies from Immuno-Biological Lab., Japan. The other authors declare that they have no conflicts of interest.

## Declaration of generative AI and AI-assisted technologies in the writing process

During the preparation of this work, we used ChatGPT 4.0 in order to improve the readability and language of the manuscript. After using this tool/service, we reviewed and edited the content as needed.

## STAR★Methods

### Key resources table

**Table:** 

REAGENT or RESOURCE	SOURCE	IDENTIFIER
**Antibodies**		

Goat polyclonal anti-Lyve-1	R&D system	AF2125; RRID:AB_2297188
Rat monoclonal anti-F4/80 (CI:A3-1)	Abcam	ab6640; RRID:AB_1140040
Rabbit monoclonal anti-CD4 (EPR19514)	Abcam	ab183685; RRID:AB_2686917
Rabbit polyclonal anti-GFP	Abcam	ab6556; RRID:AB_305564
Goat polyclonal anti-CD31	R&D system	AF3628; RRID:AB_2161028
Mouse monoclonal anti-Tenascin-C (4F10TT)	Immuno-Biological Laboratories	10337; RRID:AB_2341283
Rabbit polyclonal anti-Tenascin-C	Our lab	N/A
Rat monoclonal anti-Ki-67 (SolA15)	eBioscience	14-5698-82; RRID:AB_10854564
Rabbit monoclonal anti-CD4 (EPR19514)	Abcam	ab183685; RRID:AB_2686917
Mouse monoclonal anti-Ki-67 (MIB-1)	DAKO	M7240; RRID:AB_2142367
Rabbit polyclonal anti-cleaved Caspase-3 (Asp175)	CST	9661; RRID:AB_2341188
Rabbit monoclonal anti-integrin αV (EPR16800)	Abcam	ab179475; RRID:AB_2716738
Mouse monoclonal anti-integrin αV (P2W7)	R&D system	MAB1219; RRID:AB_357539
Mouse monoclonal anti-integrin β1 (12G10)	Abcam	Ab30394; RRID:AB_775726
Mouse monoclonal anti-integrin β1 (P5D2)	R&D system	MAB17781; RRID:AB_2129940
Mouse monoclonal anti-α-tubulin (DM1A)	Cedarlane	CTL9002; RRID:AB_3665220
Rabbit monoclonal anti-phospho TGFβ Receptor II (phospho S225) (EPR12198)	Abcam	ab183037; RRID:AB_3094566
Rabbit polyclonal anti-TGFβ Receptor	Abcam	ab186838; RRID:AB_2728775
Rabbit monoclonal anti-phospho p38 MAPK (Thr180/Tyr182) (D3F9)	CST	4511; RRID:AB_2139682
Rabbit monoclonal anti-p38 MAPK (D13E1)	CST	8690; RRID:AB_10999090
Rabbit monoclonal anti-phospho ATF-2 (Thr71) (11G2)	CST	5112; RRID:AB_560873
Rabbit monoclonal anti-phospho ATF-2 (Thr71) (E268)	CST	ab32019; RRID:AB_725567
Rabbit monoclonal anti-ATF-2 (D4L2X)	CST	35031; RRID:AB_2799069
Rabbit monoclonal anti-phospho MKK3 (Ser189)/MKK6 (Ser207) (D8E9)	CST	12280; RRID:AB_2797868
Mouse monoclonal anti-MKK3/MKK6	R&D system	MAB2514; RRID:AB_2235050
Rabbit monoclonal anti-phospho TAK1 (Ser439) (EPR2863)	Abcam	ab109404; RRID:AB_10860641
Rabbit monoclonal anti-TAK1 (D94D7)	CST	5206; RRID:AB_10694079
Rabbit polyclonal anti-phospho Smad2 (Ser465/467)	CST	3101; RRID:AB_331673
Rabbit monoclonal anti-Smad2 (86F7)	CST	3122; RRID:AB_823638
Rabbit monoclonal anti-phospho Smad3 (Ser423/425) (C25A9)	CST	9520; RRID:AB_2193207
Rabbit monoclonal anti-Smad3 (C67H9)	CST	9523; RRID:AB_2193182
Anti-mouse IgG (H + L), F(ab')2 Fragment (Alexa Fluor® 555 Conjugate)	CST	4409; RRID:AB_1904022
Goat anti-Rat IgG (H + L) Cross-Adsorbed Secondary Antibody, Alexa Fluor™ 546	Thermo	A-11081; RRID:AB_2534125
F(ab')2-Goat anti-Rabbit IgG (H + L) Cross-Adsorbed Secondary Antibody, Alexa Fluor™ 546	Thermo	A-11071; RRID:AB_2534115
Goat Anti-Rabbit IgG (H + L) Antibody, Alexa Fluor 488 Conjugated	Thermo	A-11008; RRID:AB_143165
Goat Anti-Rat IgG H&L (Alexa Fluor® 594)	Abcam	ab150160; RRID:AB_2756445
Donkey Anti-Mouse IgG H&L (Alexa Fluor® 555) preadsorbed	Abcam	ab150110; RRID:AB_2783637
Donkey Anti-Rabbit IgG H&L (Alexa Fluor® 555)	Abcam	ab150074; RRID:AB_2636997
Donkey Anti-Rat IgG H&L (Alexa Fluor® 555) preadsorbed	Abcam	ab150154; RRID:AB_2813834
Donkey Anti-Goat IgG H&L (Alexa Fluor® 647) preadsorbed	Abcam	ab150135; RRID:AB_2687955
Donkey Anti-Goat IgG H&L (Alexa Fluor® 488)	Abcam	ab150129; RRID:AB_2687506
Donkey Anti-Rat IgG H&L (Alexa Fluor® 647) preadsorbed	Abcam	ab150155; RRID:AB_2813835
HRP-labeled Anti-mouse IgG	Bio-Rad	1706516; RRID:AB_11125547
HRP-labeled Anti-rabbit IgG	Bio-Rad	1706515; RRID:AB_11125142

**Chemicals, peptides, and recombinant proteins**		

Tenascin-C	Our lab	N/A
Recombinant VEGF-C	Peprotech	100-20CD
Recombinant FGF2	R&D	3139-FB
Complete mini EDTA free	Roche Diagnostics	6402712001
Protein G Mag Sepharose	Roche Diagnostics	4693159001
e-PAGEL (E-T520L)	ATTO	2331830
Immobilon-P Membrane	Merck Millipore	IPVH00010
PVDF Blocking Reagent for Can Get Signal	Toyobo	NYPBR01
Bovine Serum Albumin Fraction V	Roche Diagnostics	10735086001
Can Get Signal Immunoreaction Enhancer Solution	Toyobo	NKB-101T
ECL Prime Western Blotting Detection Reagents	GE Healthcare	RPN2232
Precision Plus Protein Dual Color Standards	Bio-Rad	1610374
Endothelial Cell Growth Medium MV2 kit	Promo Cell	C22121
Rhodamine Phalloidin	Thermo	R415
SB203580	Cayman Chemical	13344
(5Z)-7-Oxozeaenol	Cayman Chemical	17459
SIS3	Cayman Chemical	15945
LY2109761	Selleck Chemical	S2704
Paraformaldehyde	Merck	1040051000
Formamide	Sigma Aldrich	S4117
K-CX	Falma Corporation	CS-5151
Ethylenediaminetetraacetic acid	Fujifilm wako	A10713
Dako Target Retrieval Solution (10×)	Dako	S1699
DAPI	Dojindo	342–07431
Thyoglycollate	Sigma Aldrich	70157
Donkey serum	Abcam	ab7475
Goat Serum	Gibco	16210064
3M Tegaderm Transparent Film Roll	3M Japana	16002JP
Dormycin Ointment	Zeria Pharmaceutical	N/A
Coring Matrigel Membrane Matrix (Growth Factor Reduced)	Corning	354230
Collagen Gel Culturing Kit	Nitta Gelatin Inc	638–00781
Rneasy Plus Mini Kit	QIAGEN	74134
Transcriptor First Strand cDNA synthesis Kit	Roche Diagnostics	4897030001

**Critical commercial assays**		

RT^2^ Profiler™ PCR Array Human Angiogenesis	QIAGEN	PAHS-024Z
CellTiter96 Aqueous One Solution Cell Proliferation Assay (MTS)	Promega	G3580

**Experimental models: Cell lines**		

Human Dermal Lymphatic Endothelial Cells (HDLECs)	Promo Cell	C-12217

**Oligonucleotides**		

RT^2^ qPCR Primer Assay for Human GAPDH	QIAGEN	PPH00150F
RT^2^ qPCR Primer Assay for Human ITGAV	QIAGEN	PPH00628C

**Software and algorithms**		

ImageJ	ImageJ	https://imagej.nih.gov/ij/
Graphpad Prism	GraphPad Software	N/A
Database for Annotation, Visualization, and Integrated Discovery (DAVID)	DAVID	https://davidbioinformatics.nih.gov/

### Experimental model and study participant details

#### Cells

We cultured primary human dermal lymphatic endothelial cells (HDLECs) isolated from human adult skin (PromoCell C-12217) with endothelial cell growth medium MV2 (PromoCell D12026) at 37°C in a 5% CO_2_ incubator.

#### Mice

We used 8 to 10-week-old male or female BALB/c and WT and *TNC* ^*−/−*^ mice littermates in this study and performed all animal experiments according to the Institutional Animal Use and Care Guidelines with approval by the Animal Ethics Review Committee of Mie University (2019-24). RICKN BRC provided GFP mice through the National Bio-Resource Project of the MEXT (Japan).^60^ Mice were housed and maintained in a temperature- and humidity-controlled environment.

### Method details

#### Animal model of tail lymphedema

We used 8 to 10-week-old male WT and *TNC* ^*−/−*^ mice for the tail lymphedema model. After mice were anesthetized with isoflurane, we made a 2-mm-wide circumferential incision of tail skin through the dermis, 10 mm distal to the tail base, to remove dermal lymphatic vessels without injuring blood vessels and tendons. We covered the wounds with topical antibiotic cream (Dolmycin Ointment; Zeria Pharmaceutical, Tokyo, Japan) to prevent infection. The diameter of the tail was measured with a digital caliper. In some experiments, we covered the wounds with collagen (3 mg/mL; Nitta Gelatin Inc., Osaka, Japan) containing TNC (100 μg/mL) or not, and wrapped with polyurethane film (3M Tegaderm Transparent Film Roll; 3M Japan, Tokyo, Japan) after gelation. In addition, 100 μL of PBS alone or PBS containing TNC (50 μg/mL) was intradermally injected 30 mm distal to the wound three times a week 5 days after surgery. We used TNC purified from the supernatant of the human U251 glioma cell line.^33^^,^^61^

#### Histology and immunohistochemistry

We fixed the samples in 4% paraformaldehyde (PFA) (Merck, Darmstadt, German) at 4°C for 24 h, embedded the samples in paraffin blocks, and sectioned the tissue block at a thickness of 4 μm. We used ethylenediaminetetraacetic acid (EDTA) or K-CX (Falma Corporation, Tokyo, Japan) for the decalcification of tail tissues before embedded in paraffin. Sections were stained with hematoxylin and eosin (HE). We performed heat-mediated antigen retrieval for immunohistochemistry using Dako Target Retrieval Solution (pH 6.0; S1699; Dako Japan, Tokyo, Japan) at 121°C for 1 min. After blocking the sections with 10% goat (Gibco, Invitrogen Corporation, Grand Island, NY) or donkey serum (Abcam, Cambridge, UK) at room temperature for 1 h, we incubated the sections with primary antibodies at 4°C overnight. Subsequently, sections were incubated with the appropriate secondary antibodies at room temperature for 2 h, followed by the staining with DAPI (Dojindo, Kumamoto, Japan) at room temperature for 10 min. All primary or secondary antibodies were purchased from commercial sources, except for the anti-TNC antibody raised in our laboratory.^62^ We took images with a cooled charge-coupled device camera (Olympus, Tokyo, Japan) or Keyence BZ-X710 (Keyence, Osaka, Japan). We used 3–4 randomly selected fields per animal for all histological quantification, except for the analysis of whole ear imaging. We used one whole image of the ear for the analysis.

#### Measurements of TNC concentrations in tail tissue fluid

We collected the tissue interstitial fluid by cutting off tail tissue at edematous sites and centrifuged it at 500 × g at 4°C for 5 min to remove debris. The concentration of TNC in the supernatant was measured using an ELISA kit (Immuno-Biological Laboratories, Gunma, Japan) according to the manufacturer’s instructions.

#### Analysis of lymphatic fluid drainage function of tissue fluid

We used EB dye for the evaluation of the lymphatic drainage function of tissue fluid. We injected 20 μL of 4% EB dye (Sigma Aldrich, St. Louis, MO) intradermally 10 mm from the tip of the tail. Two hours after injection, we harvested the bilateral drainage lymph nodes and incubated them in formamide (Sigma Aldrich) at 50°C for 24 h to extract EB dye from lymph nodes. The concentration of EB dye in PFA was measured using a microplate reader (Infinite F50, TECAN, Switzerland) at OD 630 nm.

#### Analysis of the lymphatic drainage function of inflammatory cells

We used GFP+ inflammatory cells for the evaluation of the lymphatic drainage function of inflammatory cells. We intraperitoneally injected 1 mL of thioglycollate solution (30 mg/ml in PBS) (Sigma-Aldrich) into GFP mice for 3 days to induce peritonitis and collected the GFP+ inflammatory cells from ascites. Approximately 1,000,000 cells/20 μL PBS were intradermally injected 20 mm distal to incision lesions without pressure. We harvested bilateral drainage lymph nodes 16 h after the injection and fixed them with PFA followed by embedding in paraffin. After staining with the GFP antibody, GFP+ cells in the lymph nodes were counted in 3 randomly selected fields per lymph node under a fluorescence microscope.

#### Animal models for zymosan-induced peritonitis

To induce peritonitis, we intraperitoneally injected zymosan solution (0.5 mg/ml in PBS) (Sigma-Aldrich, Munich, Germany) into female BALB/c mice and *TNC* ^*−/−*^ mice (8–10 weeks old) for five days. For the histological analysis, we collected the diaphragm of treated mice 23 days after the initial injection of Zymosan.

#### Matrigel plug assay

We injected growth factor-reduced Matrigel (250 μL) (Corning, New York, USA) containing FGF-2 (4 μg/mL; R&D 234-FSE) to stimulate lymphangiogenesis with or without TNC (50 μg/mL) into the backs of WT mice and *TNC* ^*−/−*^ mice (10–12 weeks old). We harvested the Matrigel 2 weeks after injection, fixed them in 4% PFA, and embedded them in paraffin for histological analysis.

#### Ear sponge assay

The gelatin sponge (GeLfoam, Pfizer) was cut into small pieces and soaked in serum-free DMEM containing VEGF-C (1 μg/mL; ProteinTech) with or without TNC (50 μg/mL) by adding a 20-μL drop on the top of the sponge. Sponges were incubated at 37°C for 30 min and embedded in cold interstitial type I collagen solution (2 mg/mL) with or without TNC (50 μg/mL). Sponges were immediately transferred into new wells and re-incubated at 37°C for 30 min to allow complete gel polymerization. Ten-week-old BALB/c male mice and *TNC* ^*−/−*^ male mice were anesthetized with isoflurane and a small horizontal incision was performed in the basal, external, and central parts of the mouse ear. The external mouse ear skin layer was smoothly detached from cartilage making a hole of 5 mm^2^ and a gelatin sponge was introduced deeper into the hole. Once the sponge was placed inside the ear, a suture point was made. Eighteen days after surgery, the ears were excised and embedded in paraffin after fixation with 4% PFA.

#### Cell culture and reagents

We cultured HDLECs, purchased from Promo Cell (Promo Cell, Heidelberg, Germany), in endothelial cell growth medium MV2 (Promo Cell) containing 5% FBS. All experiments were performed between passages 4 and 10 in the present study. We added TNC (10 μg/mL) to the medium after replacing the medium with endothelial cell growth medium MV2 containing 0.5% FBS. In some experiments, we used kinase inhibitors, including LY2109761 (TGF-β receptor inhibitor; 2 μM), SIS3 (SMAD3 inhibitor; 2 μM), 5Z-7-Oxozeaenol inhibitor (7-Oz) (TAK1 inhibitor; 100 nM), and SB203580 (p38 MAPK inhibitor; 2 μM), to examine the signaling cascade associated with TNC. Each inhibitor was added 1 h before the addition of TNC.

#### MTS assay

We seeded 3,000 cells of HDLECs into a 96-well plate (3,000 cells/well) and cultured the cells in a serum-starved medium with or without TNC for 48 h. The viability of the cells was evaluated by MTS incorporation assays using the Aqueous One Solution Cell Proliferation Assay (Promega, Madison, WI) according to the manufacturer’s instructions. The absorbance of formazan dye was measured using a microplate reader at 492 nm.

#### Tube formation assay

We added 250 μL of Matrigel (growth factor reduced) containing PBS or TNC (1, 3, 10 μg/mL) into a 24-well plate and incubated the plate at 37C for 30 min to solidify Matrigel. After HDLECs were incubated with or without TNC (1, 3, 10 μg/mL) at 37°C for 6 h, we seeded 125,000 cells of those cells in the 24 well plates coated with Matrigel. We took 3 images per sample after 16 h of incubation and analyzed the images using ImageJ software (NIH).

#### Spheroid sprouting assay

A total of 1.2 × 10^6^ LECs were mixed with growth medium MV2 supplemented with 2% FBS containing 0.24% methylcellulose (Sigma) and allowed to aggregate in round-bottom 96-well plates for 12 to 24 h (3,000 cells per spheroid). Spheroids were subsequently embedded in type 1 collagen (Nitta Gelatin, Osaka, Japan) with or without TNC (20 μg/mL) and incubated for an additional 16 h. We fixed them in 4% PFA and incubated the samples with rhodamine-phalloidin (Cytoskeleton) and DAPI. Images were observed under a Keyence BZ-X700 microscope and analyzed using ImageJ software (NIH).

#### Immunofluorescence staining of cells

HDLECs, grown on micro cover glasses (Matsunami, Kishiwada, Japan), were fixed and permeabilized in 4% PFA containing 0.2% Triton X- for 10 min. We used 10% goat or donkey serum for blocking at room temperature for 1 h, and then cells were incubated with primary antibodies at 4°C overnight. The following day, cells were incubated with secondary antibodies at room temperature for 1 h and DAPI (Dojindo) at room temperature for 10 min before embedding in mounting medium (Invitrogen). Proliferating cells, defined as ki-67+ cells, were counted in 4 randomly selected fields per sample. Apoptotic cells, defined as cleaved caspase-3-positive cells, were counted in 8 randomly selected fields per sample. To assess the ratio of phospho-ATF2-positive nuclei, 200 cells were analyzed per sample.

#### Western blotting

Cells were lysed with lysis buffer (1% SDS and 50 mM Tris–HCl buffer (pH 6.8)) with or without 5% 2-mercaptoethanol, and heated at 98°C for 5 min. We used lysis buffer without 2-mercaptoethanol for the detection of active β1 integrin. We performed electrophoresis using 5%–20% polyacrylamide gradient gels (e-PAGEL; E-T520L, ATTO, Tokyo, Japan) at 20 mA for 60 min or at 40 mA for 80 min. Proteins were electrically transferred to Immobilon membranes (Merck Millipore, Carrigtwohill, Ireland) at 400 mA for 2 h. Membranes were incubated with blocking buffer, such as PVDF Blocking Reagent (Toyobo, Osaka, Japan) or 2% bovine serum albumin (BSA) (Bovine Serum Albumin Fraction V, Roche Diagnostics, Manheim, Germany) in TBS containing 0.05% Tween 20 (TBS-T) at room temperature for 1 h. Membranes were probed with the primary antibody at 4°C overnight, followed by an incubation with a peroxidase-labeled secondary antibody at room temperature for 2 h. Primary and secondary antibodies were diluted in Can Get Signal Immunoreaction Enhancer Solution (Toyobo) or TBS-T containing 2% BSA. The specific binding of an antibody to a membrane protein was detected using the ECL Prime Western Blotting Detection Reagent (GE Healthcare, Waukesha, WI). We used Precision Plus Protein Dual Color Standards (Bio-Rad Laboratories) as molecular weight markers. We used ImageJ for the quantification of band intensities. We used α-tubulin as an internal control. When we quantified the phosphorylation level of the genes, we used the total protein as a control. For the quantification, we used ImageJ and the values were normalized to intensities of α-tubulin bands.

#### Immunoprecipitation

Immunoprecipitation was performed as previously reported.^33^ Briefly, cells were lysed in lysis buffer (1% Nonidet P-40, 20 mM Tris–HCl buffer (pH 7.5), 150 mmol/L NaCl, 1 mmol/L CaCl2, and 1 mmol/L MgCl2) with protease inhibitors (Complete mini EDTA free; Roche Diagnostics), and lysates were centrifuged at 15,000 × g at 4°C for 20 min. The supernatants were pre-cleared with protein G beads (Protein G Mag Sepharose, GE Healthcare) for 30 min followed by incubation with the primary antibodies, and complexes were precipitated using protein G beads (GE Healthcare). After the beads had been boiled with sample buffer, immunoblotting was performed.

#### Quantitative reverse-transcription PCR and qiagen RT profiler PCR array

Total RNA, extracted from treated cells using an RNeasy Plus Mini Kit (Qiagen, Valencia, CA), was reverse-transcribed into cDNA by a Transcriptor First Strand cDNA Synthesis Kit (Roche Diagnostics) with an anchored-oligo (dT)18 primer. Quantitative PCR was performed using a LightCycler real-time PCR instrument with a FastStart Essential DNA Green Master kit (Roche Diagnostics). After initial denaturation (at 95°C for 10 min), 55 cycles (at 95°C for 10 s, 60°C for 10 s, and 72°C for 10 s) of amplification were performed. Primers for the target genes were purchased from Qiagen (Valencia, CA). The expression of GAPDH was used for normalization. For the PCR array experiment, we treated the HDLECs with TNC for 24 h and examined the expression of angiogenic-related genes using RT2 Profile PCR Arrays (PAHS-024Z, Qiagen, USA).

#### Microarray data information

We downloaded the 6 samples, containing 3 arrays of normal tail skin (no intervention) and 3 arrays of injured tail skin with lymphedema (14 days after surgery), from the GSE4333 expression profile and correlated information from the NCBI-Gene Expression Omnibus website. We analyzed those 6 samples to identify DEGs between the normal and lymphedema groups. DEGs were defined as the fold change (FC) cut-off was >2 or 0.5>, and the adjusted *p* value cut-off was <0.05. We used the Database for Annotation, Visualization, and Integrated Discovery (DAVID) to assess the GO using the official gene symbol and species information (Mus musculus). GO analysis terms were classified into biological process (BP), cellular component (CC), and cellular process (CP) categories.

#### Reagent and antibodies

The reagents and antibodies used in the present study are listed in the key resource table.

### Quantification and statistical analysis

Statistical analyses were performed using GraphPad Prism software. The significance of differences was calculated using an unpaired t-test, a one-way analysis of variance followed by Tukey’s multiple comparisons, or a two-way analysis of variance. *p* < 0.05 was considered to be statistically significant.

## Supplemental information

Document S1. Figures S1–S5

Table S1. Top pathways enriched in lymphedematous mouse tail tissues compared to normal tail tissues, related to Figure 1

Table S2. Gene expression in lymphedematous mouse tail tissues compared to normal tail tissues, related to Figure 1

Table S3. Altered expression of angiogenesis-related genes in lymphatic endothelial cells treated with Tenascin-C, related to Figure 5
