# Supplementary material for: Negative regulation of lymphangiogenesis by Tenascin-C delays the resolution of inflammation
Source: iScience. 2025 Jan 6;28(2):111756. doi: 10.1016/j.isci.2025.111756 (PMC11803235; doi:10.1016/j.isci.2025.111756)
Supplement: Document S1. Figures S1–S5 [file mmc1.pdf]

iScience, Volume *28*

Supplemental information

Negative regulation of lymphangiogenesis by Tenascin-C delays the resolution of inflammation

Daisuke Katoh, Yoshiyuki Senga, Kento Mizutani, Kazuaki Maruyama, Daishi Yamakawa, Tadashi Yamamuro, Michiaki Hiroe, Keiichi Yamanaka, Akihiro Sudo, Naoyuki Katayama, Toshimichi Yoshida, and Kyoko Imanaka-Yoshida

Figure S1

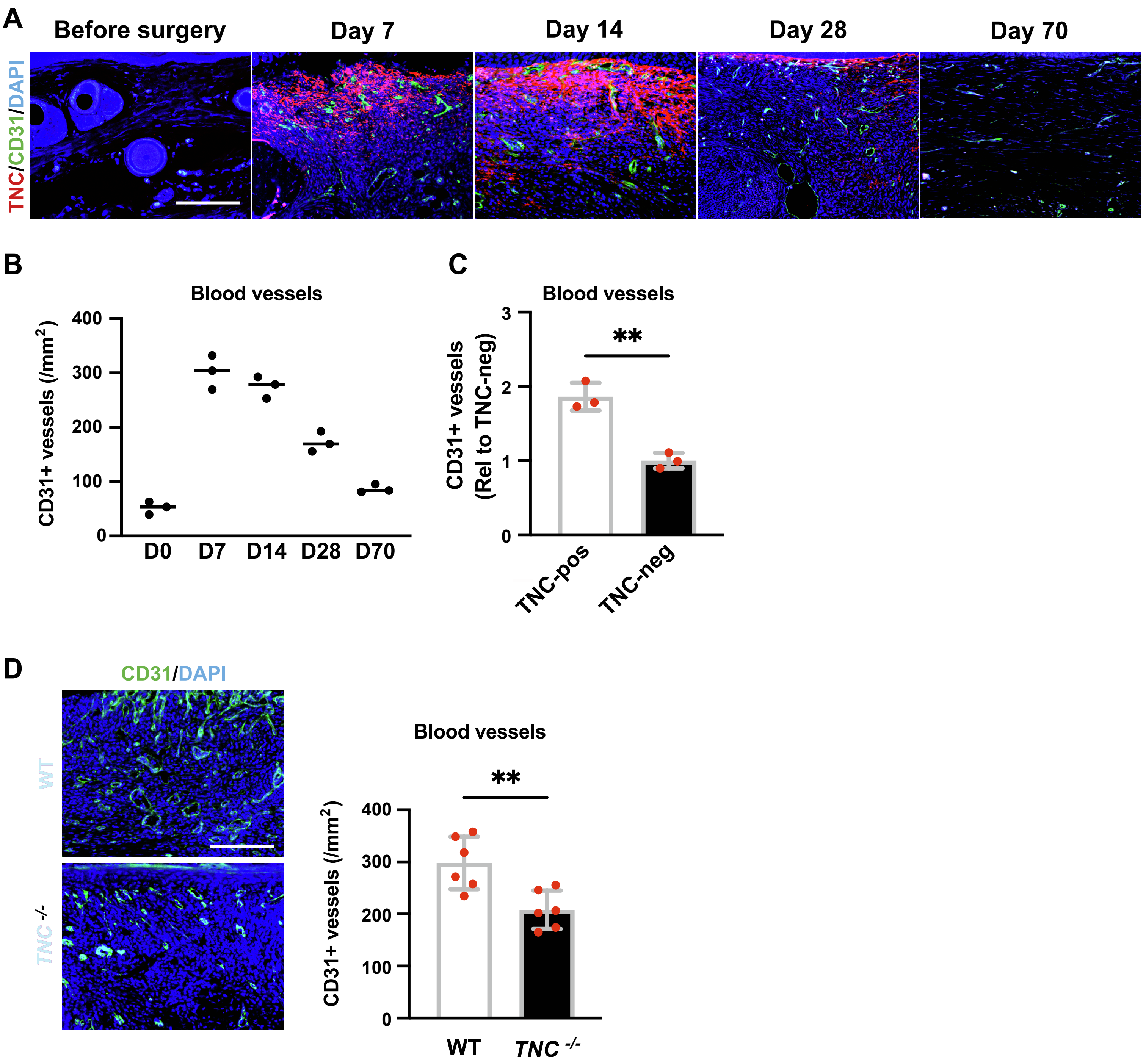
A
Before surgery
Day 7
Day 14
Day 28
Day 70
TNC/CD31/DAPI
B
Blood vessels
CD31+ vessels (/mm2)
400
300
200
100
0
D0
D7
D14
D28
D70
C
Blood vessels
CD31+ vessels
(Rel to TNC-neg)
3
2
1
0
**
TNC-pos
TNC-neg
D
CD31/DAPI
WT
TNC -/-
Blood vessels
**
CD31+ vessels (/mm2)
400
300
200
100
0
WT
TNC -/-

Supplemental Figure 1: Tenascin-C promotes angiogenesis during lymphedema.

(A–C) Analysis of the relationship between TNC expression and blood vessels in tail lymphedema mouse model. The chronological histology of immunofluorescent images of TNC (red) and CD31 (green) from day 0 (before surgery) to 70 dpi (A). A plot of the number of CD31+ blood vessels in tail tissues. n = 3 for each time point (B). Quantitative analysis of the number of blood vessels in the area with or without TNC expression at 7 dpi (C). n = 3.

(D) Assessment of the number of blood vessels in injured tail tissues of WT and *TNC* $^{-/-}$ mice. Representative immunofluorescence images of tail tissues using markers for blood vessels (CD31; green) (D, left), and quantification of the number of blood vessels (D, right). n = 6 in each group.

Each dot represents a value obtained from one sample. Data are presented as means ± SD, Scale bars: 250 µm (A and D). **P<0.01 (an unpaired t-test) (C and D).

Figure S2

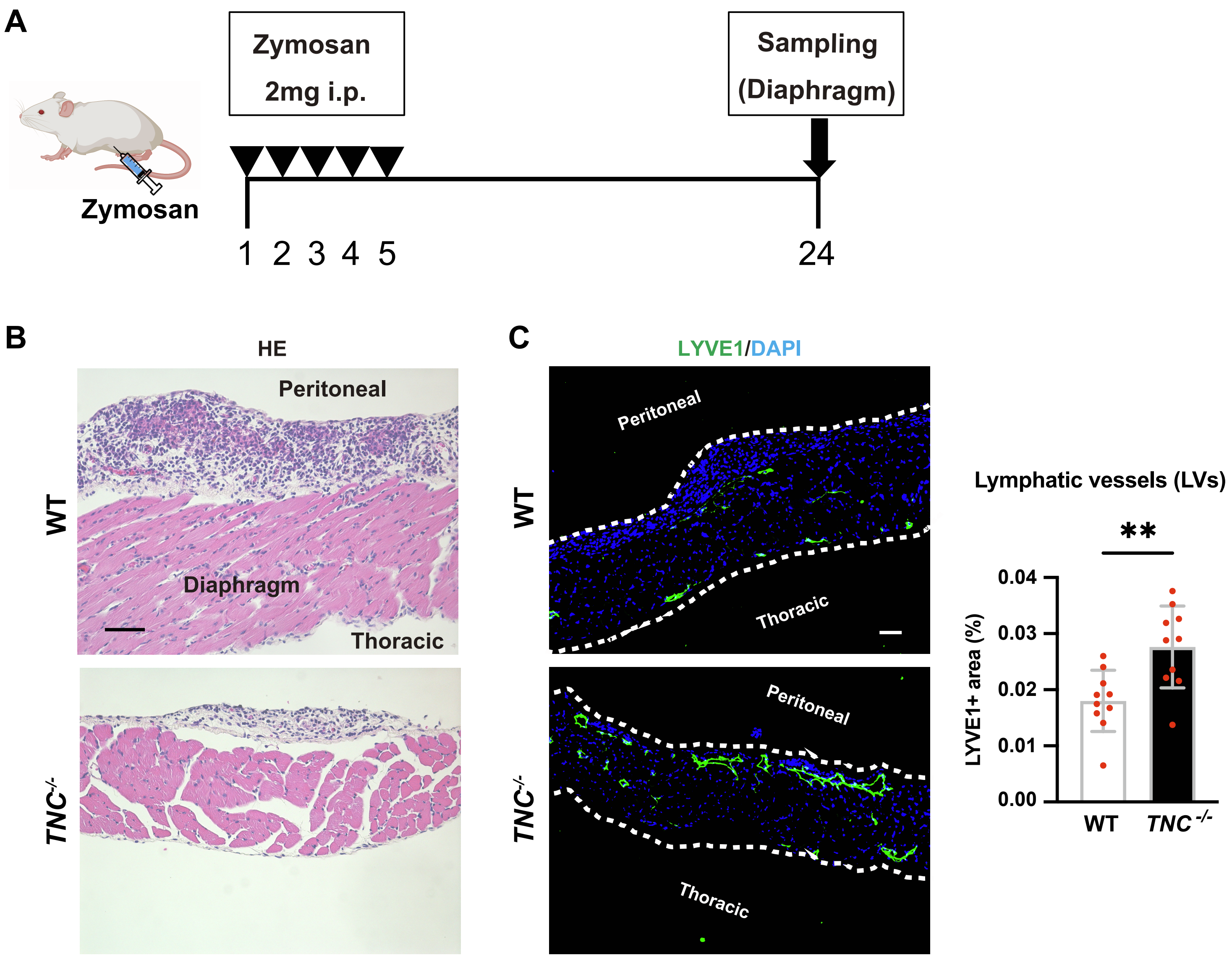
A
Zymosan
2mg i.p.
Sampling
(Diaphragm)
Zymosan
1 2 3 4 5
24
B
HE
Peritoneal
WT
Diaphragm
Thoracic
TNC-/-
C
LYVE1/DAPI
Peritoneal
WT
Thoracic
Peritoneal
TNC-/-
Thoracic
Lymphatic vessels (LVs)
**
LYVE1+ area (%)
0.04
0.03
0.02
0.01
0.00
WT
TNC -/-

Supplemental Figure 2: Deletion of tenascin-C promotes lymphangiogenesis in a zymosan-induced peritonitis model.

(A–C) Comparison of the growth of lymphatic vessels in WT and *TNC* $^{-/-}$ mice treated with zymosan. Schematic illustration of a zymosan-induced peritonitis model: WT and *TNC* $^{-/-}$ mice received a peritoneal injection of zymosan for 5 days and were sacrificed 23 days after the initial injection of zymosan (A). Representative histology (B) and immunofluorescent images of LYVE1 (green) in the diaphragm of WT and *TNC* $^{-/-}$ mice (C). Quantitative evaluation of lymphatic vessel density (C, right). n=10 in each group.

Each dot represents a value obtained from one sample. Data are presented as means ± SD, Scale bars: 100 µm. **P<0.01 (an unpaired t-test).

Figure S3

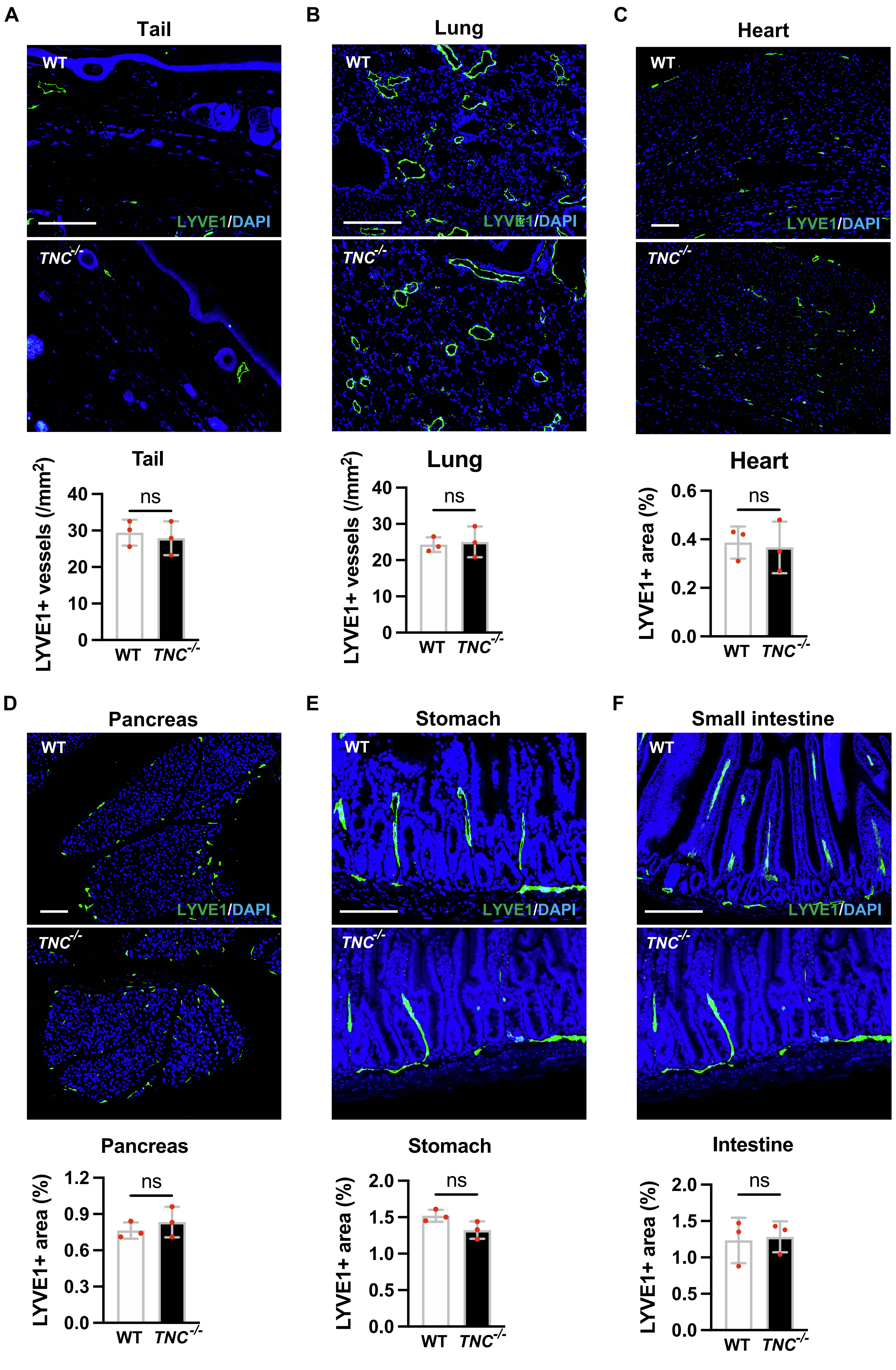
A
Tail
WT
LYVE1/DAPI
TNC-/-
B
Lung
WT
LYVE1/DAPI
TNC-/-
C
Heart
WT
LYVE1/DAPI
TNC-/-
Tail
LYVE1+ vessels (/mm2)
40
30
20
10
0
ns
WT
TNC-/-
Lung
LYVE1+ vessels (/mm2)
40
30
20
10
0
ns
WT
TNC-/-
Heart
LYVE1+ area (%)
0.6
0.4
0.2
0.0
ns
WT
TNC-/-
D
Pancreas
WT
LYVE1/DAPI
TNC-/-
E
Stomach
WT
LYVE1/DAPI
TNC-/-
F
Small intestine
WT
LYVE1/DAPI
TNC-/-
Pancreas
LYVE1+ area (%)
1.2
0.9
0.6
0.3
0.0
ns
WT
TNC-/-
Stomach
LYVE1+ area (%)
2.0
1.5
1.0
0.5
0.0
ns
WT
TNC-/-
Intestine
LYVE1+ area (%)
2.0
1.5
1.0
0.5
0.0
ns
WT
TNC-/-

Supplemental Figure 3: Tenascin-C does not affect the normal development of lymphatic vessels in various tissues.

(A–F) Comparison of lymphatic vessels of various tissues in WT and *TNC* $^{-/-}$ mice. Immunofluorescent imaging for LYVE1 (green) (upper) and the quantitative evaluation of lymphatic vessel density between WT and *TNC* $^{-/-}$ mice (lower) in the tail (A), lung (B), heart (C), pancreas (D), stomach (E), and small intestine (F). n=3 in each group.

Each dot represents a value obtained from one sample. Data are presented as means ± SD, Scale bars: 100 µm. ns: not significant (an unpaired t-test).

Figure S4

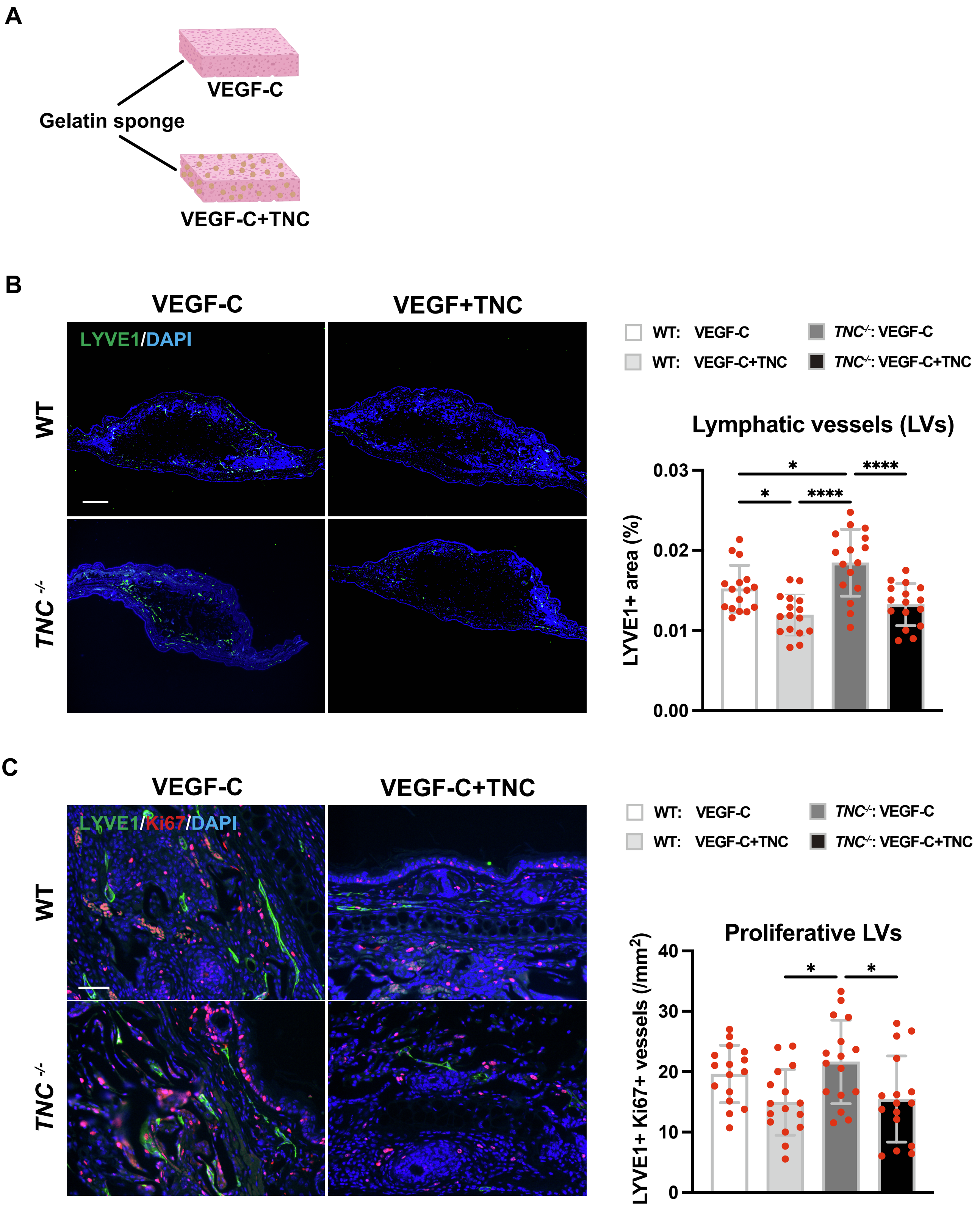
A
VEGF-C
Gelatin sponge
VEGF-C+TNC
B
VEGF-C
VEGF+TNC
LYVE1/DAPI
WT
TNC -/-
WT: VEGF-C
WT: VEGF-C+TNC
TNC-/-: VEGF-C
TNC-/-: VEGF-C+TNC
Lymphatic vessels (LVs)
LYVE1+ area (%)
0.00
0.01
0.02
0.03
*
****
C
VEGF-C
VEGF-C+TNC
LYVE1/Ki67/DAPI
WT
TNC -/-
WT: VEGF-C
WT: VEGF-C+TNC
TNC-/-: VEGF-C
TNC-/-: VEGF-C+TNC
Proliferative LVs
LYVE1+ Ki67+ vessels (/mm2)
0
10
20
30
40
*

Supplemental Figure 4: Tenascin-C inhibits lymphangiogenesis induced by VEGF-C

(A-C) Analysis of the effects of TNC on VEGF-C induced lymphangiogenesis. Schematic illustration of ear sponge assay using gelatin sponge soaked with VEGF-C (1 μg/ml) alone or with TNC (50 μg/ml) implanted between the mouse ear skin layers (A). Representative immunofluorescent images for LYVE1 (green) of the whole ear tissues containing gelatin sponge (B, left) and the quantification of lymphatic vessel density (B, right). Representative immunofluorescent images for LYVE1 (green) and Ki67 (red) (C, left) and the quantification of proliferative lymphatic vessels (C, right). n=16 in each group.

Each dot represents a value obtained from one sample. Data are presented as mean ± SD. Scale bars: 1000 μm (B), 150 μm (C). *$P < 0.05$, ****$P < 0.0001$ (a one-way ANOVA with the post-hoc Tukey's test).

Figure S5

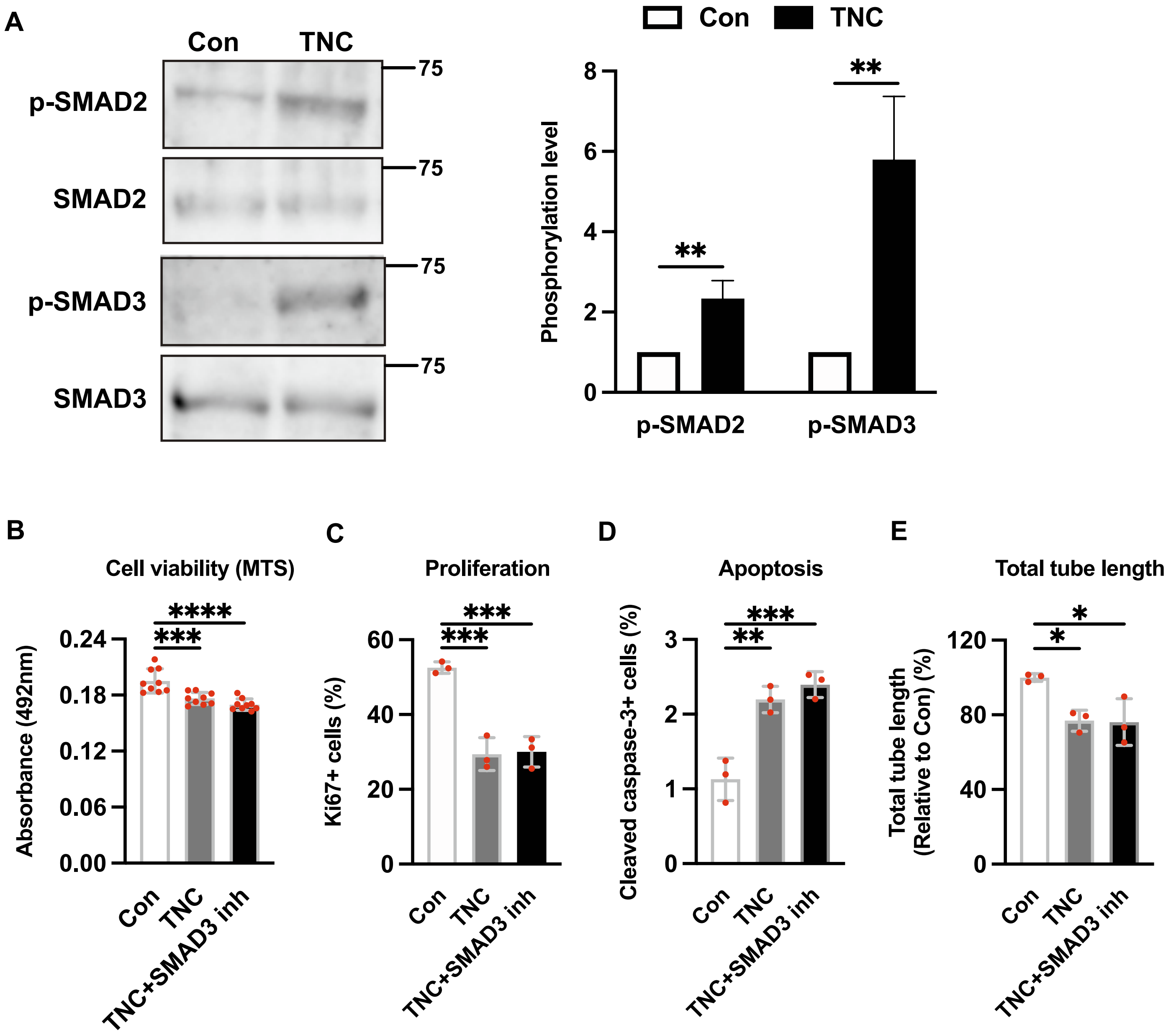
A
Con
TNC
p-SMAD2
SMAD2
p-SMAD3
SMAD3
75
75
75
75
Con
TNC
Phosphorylation level
8
6
4
2
0
**
**
p-SMAD2
p-SMAD3
B
Cell viability (MTS)
Absorbance (492nm)
0.24
0.18
0.12
0.06
0.00
****
***
Con
TNC
TNC+SMAD3 inh
C
Proliferation
Ki67+ cells (%)
60
40
20
0
***
***
Con
TNC
TNC+SMAD3 inh
D
Apoptosis
Cleaved caspase-3+ cells (%)
3
2
1
0
***
**
Con
TNC
TNC+SMAD3 inh
E
Total tube length
Total tube length
(Relative to Con) (%)
120
80
40
0
*
*
Con
TNC
TNC+SMAD3 inh

Supplemental Figure 5: SMAD activation does not involve TNC-induced negative effects on HDLECs

(A) Analysis of the effects of TNC on the phosphorylation of SMAD pathways. Immunoblot analysis of the phosphorylation of SMAD2 and SMAD3 after treatment with TNC for one hour (A, left), and quantification of phospho-SMAD2 and phospho-SMAD3 (A, right).

(B–E) Analysis of the inhibitor of the SMAD3 inhibitor (SIS3; 2μM) on TNC-induced negative effects of HDLECs. Quantification of cell viability evaluated by the MTS assay (B), the number of the ki67 positive proliferative cells (C), the number of the cleaved caspase-3 positive apoptotic cells (D), and tube formation ability by total tube length measurements (E) in HDLECs treated with TNC in the presence or absence of a SMAD3 inhibitor.

Each dot represents a value obtained from one sample. Data are presented as mean ± SD. *P < 0.05, **P < 0.01, ***P < 0.001 (an unpaired t-test (A), and a one-way ANOVA with the post-hoc Tukey's test (B–E)).
